# Foot health status and associated characteristics of nursing students: a cross-sectional study

**DOI:** 10.1186/s12912-025-03327-y

**Published:** 2025-07-01

**Authors:** Na-Geong Kim, Hye-Ryeon Park

**Affiliations:** 1 Department of Nursing, Busan Health University, 16, Sari-ro 55beon-gil, Saha-gu, Busan, Republic of Korea; 2 Department of Nursing, Kyungil University, 50, Gamasil-gil, Hayang- eup, Gyeongsan-si, Gyeongsangbuk-do Republic of Korea

**Keywords:** Students, Nursing, Stress psychological, Foot, Health status

## Abstract

**Background:**

Nursing students frequently experience prolonged standing during clinical practice, which can lead to foot health disorders. Despite its critical importance for overall well-being and future job performance, foot health among nursing students remains understudied. This study aimed to examine foot health status and associated characteristics among nursing students.

**Methods:**

A cross-sectional online survey was conducted with 184 nursing students. The survey collected data on general characteristics, foot-related disease and methods for relieving foot pain, stress, foot health status. T-tests and ANOVA were employed to examine stress and foot health status according to participants’ general characteristics.

**Results:**

Approximately 93.5% of nursing students reported foot discomfort, with prevalent foot-related diseases including toenail disorders, tinea pedis, eczema, and plantar fasciitis. Stress showed statistically significant variations based on academic year (F = 3.62, *p* = .014), clinical practice period (F = 3.60, *p* = .014), foot self-care (t = 2.97, *p* = .003), and experience using foot care facilities (t = 2.28, *p* = .024). General foot health showed significant differences according to daily standing time (F = 3.74, *p* = .006), foot self-care (t = 3.57, *p* < .001), and current foot discomfort (t = 6.84, *p* < .001).

**Conclusion:**

This study comprehensively documented the extensive foot health challenges faced by nursing students. The findings underscore the urgent need to develop and implement targeted educational programs that enhance students’ awareness and self-care abilities regarding foot health. Such interventions could significantly contribute to improving the occupational health and future quality of life for nurses.

**Clinical trial number:**

Not applicable.

## Introduction

The foot is a complex structure that plays essential roles in supporting body weight and absorbing shock during movement [1], particularly crucial for healthcare professionals who spend long hours standing and walking. When foot health is compromised, it disrupts body balance, leading to postural abnormalities and functional disorders [2]. Furthermore, the foot’s structure may deform due to its inability to withstand weight-bearing [2]. These issues are especially concerning healthcare settings, where abnormal foot status can cause gait and musculoskeletal disorders (MSD) in the knees and calves [3, 4].

The impact of prolonged standing on foot health is significant, affecting both circulation and musculoskeletal function [5, 6]. The foot, often referred to as the “second heart” due to its dense concentration of peripheral blood vessels [5], is the endpoint of blood circulation and must work against gravity to return blood to the heart [6]. Poor foot health can lead to circulatory disorders, affecting overall physical well-being [7], particularly in professions requiring extended periods of standing.

Recent studies have highlighted the significant impact of foot health problems on healthcare professionals, particularly nurses and nursing students. MSD affecting the foot and ankle are common among nurses, with prevalence estimates ranging from 1.8 to 74% [8]. Nursing students, as future healthcare professionals, are regularly exposed to demanding tasks during clinical practice such as prolonged standing, lifting, and extensive ambulation within their educational curriculum, which can adversely impact their satisfaction and overall quality of life (QoL) [9]. A recent scoping review identified various foot and ankle disorders among nurses exposed to prolonged standing, emphasizing the need for early prevention and intervention [10].

The consequences of these foot health challenges are particularly significant for nursing students during their clinical practice [11]. Studies have linked musculoskeletal symptoms to an average dropout rate of 12% at the onset of nurses’ careers [9], with foot and ankle MSD being a major contributor. These conditions frequently lead to compromised physical activity and absenteeism, affecting approximately 16.7% of nurses [8]. Despite the prevalence and impact of these issues, comprehensive studies examining both foot health status and associated characteristics among nursing students are limited, particularly in diverse healthcare settings [12].

Given the significance of the physical well-being of nursing students, particularly in lower limb health, understanding the biomechanical determinants becomes critical for both their future professional trajectories and overall QoL [9]. Early assessment and intervention regarding foot health could help prevent the development of chronic problems [8]. Various factors may influence nursing students’ foot health, including general characteristics such as average daily standing time, duration of clinical practice, and other relevant factor, foot self-care, and psychological aspects such as stress [13]. Additionally, the presence of foot-related disease like toenail disorders, tinea pedis, and plantar fasciitis may impact their overall foot health status and function [14, 15]. Therefore, this study aims to comprehensively assess nursing students’ foot health status across multiple domains (foot pain, foot function, easiness of footwear choice, and general foot health) and investigate how personal, clinical practice related, and psychological factors are associated with their foot health outcomes. The specific research questions guiding this study are:

1. What is the current status of foot-related diseases, methods for relieving foot pain, stress, and foot health among nursing students?
2. How do the associated characteristics of nursing students relate to their foot health status and stress?

## Methods

### Design

This study is a cross-sectional study that examines the foot health status and identifies associated characteristics of nursing students.

### Participants

The participants of this study were nursing students from universities in B metropolitan city and K province who had clinical practice experience in healthcare settings. All participants could access the online survey platform.

The number of participants was calculated using the G*power 3.1.9.7 program. Based on one-way ANOVA, the parameters were set as follows: medium effect size 0.25, significance level 0.05, power 0.80, and 4 groups. The minimum sample size required was 180 participants. Considering a dropout rate of approximately 10%, questionnaires were distributed to a total of 198 individuals. After excluding 14 incomplete responses, 184 questionnaires were used in the analysis.

### Measures

(I) General characteristics
  General characteristics were assessed using a structured self-administered questionnaire developed based on the STROBE guidelines. The questionnaire collected demographic information including gender, age, and academic year. Standing time was measured as the total daily hours spent in both static standing and ambulatory positions during clinical practice. Clinical practice period was quantified as the total accumulated weeks in healthcare settings. Footwear was collected including types of shoes worn during clinical practice, method of shoe purchase, preferred shoe type, heel height (measured in centimeters), and shoe size (using Korean standard sizing). Foot health behaviors were evaluated using questions assessing the presence or absence of foot self-care, attention to foot health, experience using foot care facilities, and current foot discomfort assessed on a 5-point Likert scale.
(II) Foot-related disease and method for relieving foot pain
  For assessing both foot-related disease and method for relieving foot pain, multiple-response questionnaires were based on literature review of conditions commonly affecting healthcare professionals. The study examined specific foot conditions including toenail disorders, tinea pedis, eczema, plantar fasciitis, callus, and other conditions. For relieving foot pain, methods including rest, stretch, try not to walk as much, limb elevation, and others were included. In both questionnaires, participants could select all applicable items and had the option to add any additional conditions or methods not listed in the original questionnaires.
(III) Stress
  This study utilized the Perceived Stress Scale (PSS) developed by Cohen et al. (1983) [16], which was translated into Korean by Park, and Seo (2010) [17]. The instrument is a continuous-type scale consisting of a 5-point Likert scale (never = 0, very often = 4) with 10 items divided into two subscales: negative perception (5 items) and positive perception (5 items). Positive items are reverse scored, with higher total scores indicating higher stress. The total score ranges from 0 to 40 points. During the original tool development, the Cronbach’s α for negative and positive perceptions was 0.77 and 0.74, respectively [16]. In this study, Cronbach’s α for negative and positive perceptions was 0.72 and 0.73, respectively.
(IV) Foot health status
  This study utilized the Foot Health Status Questionnaire (FHSQ) developed by Bennett et al. (1998) [18], which was translated into Korean by Kim (2012) [19]. The instrument was developed to assess subjective foot health status and consists of 13 items: foot pain (4 items), foot function (4 items), easiness of footwear choice (3 items), and general foot health (2 items). Each item is rated on a 5-point Likert scale. Except for items related to footwear choice, all items are reverse-coded. The total scores are calculated using the software program developed by the original authors and converted to a scale of 0-100. Lower scores indicate more negative outcomes: more severe foot pain, poorer physical foot function (indicating limitations in daily activities), greater difficulty in choosing appropriate footwear for one’s feet, and poorer subjective perception of general foot health. During the original tool development, the Cronbach’s α ranged from 0.85 to 0.88 [18]. In this study, the Cronbach’s α ranged from 0.80 to 0.92.

### Data collection and ethical considerations

This study was approved by the Institutional Review Board of Kyungil University (Approval No. 1041459-202311-HR-001-01). Data collection was conducted from January 3 to 23, 2024, focusing on nursing students with clinical practice experience.

Participant recruitment was facilitated through announcements containing QR codes that were posted in locations frequently visited by potential participants at nursing departments of universities in these regions. These QR codes directed interested students to a secure online platform. The research purpose and methods were thoroughly explained to participants, and only those who voluntarily provided informed consent by checking an agreement box could proceed to the survey. Participants were informed that they could withdraw at any time without consequences, and all data were kept anonymous. All measurement instruments were used after obtaining appropriate permissions. This study was conducted in accordance with the principles of the Declaration of Helsinki. Upon completion of the survey, participants received a small token of appreciation.

### Data analysis

The collected data were analyzed using SPSS/WIN 21.0 statistical software as follows:

(I) Descriptive statistics, including frequencies, means, and standard deviations, were used to analyze participants’ general characteristics, foot-related disease and methods for relieving foot pain, stress, and foot health status.
(II) T-tests and ANOVA were employed to examine stress and foot health status according to participants’ general characteristics, with statistical significance set at *p* < .05. Post-hoc analysis for group differences was conducted using Scheffe’s test.

## Results

### General characteristics

Female students comprised most of the sample (*n* = 150, 81.5%). The mean age of participants was 21.26 ± 6.73 years. Participants were distributed across academic years, with fourth-year students representing the largest group (*n* = 54, 29.3%). Clinical practice experience was reported by 107 participants (58.1%).

Regarding footwear during clinical practice, the most common choice was sneaker-type shoes (*n* = 67, 36.4%). Most participants obtained their clinical shoes through bulk purchase (*n* = 83, 45.1%). When asked about preferred shoe types, sneakers were most popular (*n* = 127, 69.0%). A majority of participants (*n* = 138, 75.0%) preferred heel heights less than 3 cm. More than half of the participants (*n* = 101, 54.9%) preferred shoes slightly larger than their foot size.

Regarding foot self-care practices, approximately half of the participants (*n* = 100, 54.3%) reported engaging in foot self-care, while few (*n* = 19, 10.3%) had experience using professional foot care facilities. Many participants (*n* = 172, 93.5%) reported current foot discomfort (Table 1).

**Table 1:** General characteristics of the participants (*N* = 184)

Characteristics	Categories	*n*(%)	Mean±SD
Gender	Female	150(81.5)	
	Male	34(18.5)	
Age	≤ 25	127(69.0)	21.26± 6.73
	26–30	31(16.8)	
	31–35	10(5.5)	
	≥ 36	16(8.7)	
Academic year	1st	42(22.8)	
	2nd	35(19.1)	
	3rd	53(28.8)	
	4th	54(29.3)	
Daily standing time (hr)	< 2	35(19.0)	
	2–4	40(21.7)	
	4–6	52(28.4)	
	6–8	40(21.7)	
	> 8	17(9.2)	
Clinical practice period (month)	No experience	77(41.9)	
	< 6	14(7.6)	
	< 12	44(23.9)	
	≥ 12	49(26.6)	
footwear during clinical practice ^†^	Sneaker type	67(36.4)	
	Sandal type	33(17.9)	
	Others	7(3.8)	
Method of Shoe purchase^†^	Bulk purchase	83(45.1)	
	Individual purchase	24(13.0)	
Preferred shoe type	Sneakers	127(69.0)	
	Slippers	48(26.1)	
	Others	9(4.9)	
Preferred heel height (cm)	< 3	138(75.0)	
	≥ 3	46(25.0)	
Preferred shoe size	Same	80(43.5)	
	Larger	101(54.9)	
	Smaller	3(1. 6)	
Foot self-care	Yes	100(54.3)	
	No	84(45.7)	
Attention to foot health	Low	60(32.6)	
	Moderate	97(52.7)	
	High	27(14.7)	
Experience using foot care facilities	Yes	19(10.3)	
	No	165(89.7)	
Current foot discomfort	Yes	172(93.5)	
	No	12(6.5)	

^†^No experience in clinical practice was excluded from this section

### Foot-related disease and method for relieving foot pain

The most common foot-related diseases were toenail disorders (*n* = 51, 24.8%), including ingrown toenails and nail thickening, followed by tinea pedis (*n* = 22, 10.7%), eczema (*n* = 21, 10.2%), plantar fasciitis (*n* = 20, 9.7%), plantar keratosis (*n* = 18, 8.7%), and corns (*n* = 7, 3.4%).

Regarding methods for relieving foot pain, participants most frequently reported resting their feet (*n* = 151, 26.4%), stretching exercises for foot (*n* = 110, 19.2%), try not to walk as much (*n* = 78, 13.6%), ice to the sole or heel (*n* = 65, 11.3%) (Table 2).

**Table 2:** Foot-related disease and method for relieving foot pain of participants (*N* = 184)

Foot-related disease^†^	*n*(%)
Toenail disorders (e.g. Ingrown toenails, Nail thickening)	51(24.8)
Tinea pedis (Athlete’s foot)	22(10.7)
Eczema	21(10.2)
Plantar fasciitis	20(9.7)
Callus	19(9.2)
Hallux valgus (Bunion)	18(8.7)
Plantar keratosis	18(8.7)
Flat feet	16(7.8)
Corn	7(3.4)
Achilles tendinitis	6(2.9)
Metatarsalgia	3(1.5)
Other conditions (Swelling, numbness, burning sensation)	5(2.4)

Method for Relieving Foot Pain^†^	
Rest	151(26.4)
Stretch exercises for foot	110(19.2)
Try not to walk as much	78(13.6)
Ice to the sole or heel	65(11.3)
Limb elevation	64(11.2)
Use over-the-count gel or shoe orthotics	37(6.5)
Use dietary supplements	25(4.4)
Apply a disposable patch to relieve discomfort	15(2.6)
Take a foot bath	14(2.4)
Change the shoes	7(1.2)
Lose body weight	4(0.7)
Others	3(0.5)

Duplicate response

### Stress and foot health status

The mean stress score was 27.35 ± 3.54. Regarding foot health status, the mean scores were as follows: foot pain 83.52 ± 15.21, foot function 81.64 ± 18.63, easiness of footwear choice 65.82 ± 21.32, and general foot health 70.53 ± 17.83 (Table 3).

**Table 3:** Stress and foot health status of participants (*N* = 184)

Categories	Mean±SD	Range
Stress	27.35± 3.54	17.0–40.0
Foot health status		
Foot pain	83.52± 15.21	20.0-100.0
Foot function	81.64± 18.63	20.0-100.0
Easiness of footwear choice	65.82± 21.32	20.0-100.0
General foot health	70.53± 17.83	20.0-100.0

SD = Standard deviation

### Differences of stress and foot health status by general characteristics

Significant differences in stress were observed across academic year (F = 3.62, *p* = .014), clinical practice period (F = 3.60, *p* = .014), foot self-care (t=-2.97, *p* = .003), and experience using foot care facilities (t = 2.28, *p* = .024).

Fourth-year students demonstrated higher stress (28.07 ± 3.41) than first-year students (26.40 ± 3.30). Nursing students with more than one year of clinical practice experience exhibited higher stress (28.10 ± 3.52) in comparison to those without experience (26.32 ± 3.44). Participants who practiced foot self-care by using pain relief methods reported higher stress levels (27.62 ± 4.13) than those who did not engage in such practices (27.31 ± 3.21). Additionally, participants with experience using foot care facilities recorded higher stress (29.05 ± 3.78) compared to those without such experience (27.12 ± 3.47).

Significant differences in foot pain were observed across daily standing time (F = 5.70, *p* < .001), foot self-care (t = 4.33, *p* < .001), and current foot discomfort (t=-3.25, *p* < .001). Post-hoc analysis revealed that participants with 6–8 h of daily standing time reported more severe foot pain (75.88 ± 19.87) compared to those with less than 2 h (91.86 ± 11.25). Participants who reported with foot self-care showed more foot pain (82.67 ± 18.40) compared to those who without foot self-care (84.18 ± 13.28). Those with current foot discomfort experienced more severe foot pain (70.00 ± 15.23) compared to those without (84.39 ± 14.81).

Regarding foot function, significant differences were found in relation to daily standing time (F = 5.04, *p* = .001), and foot self-care (t = 3.48, *p* < .001). Post-hoc analysis showed that participants with 4–6 h (79.33 ± 18.81) and 2–4 h (82.75 ± 16.37) of daily standing time had poorer foot function compared to those with less than 2 h (92.43 ± 12.80). Participants who reported with foot self-care showed poorer foot function (80.08 ± 20.55) compared to those who without foot self-care (83.61 ± 17.20).

Significant differences in easiness of footwear choice were observed across age (F = 2.98, *p*  =.033), daily standing time (F = 3.60, *p* = .008), and attention to foot health (F = 3.76, *p* = .025). Post-hoc analysis revealed that participants aged 36 and above reported more difficulty in footwear choice (52.50 ± 21.20) compared to those 25 years old or younger (68.19 ± 21.19). Participants with 6–8 h of daily standing time experienced more difficulty (57.00 ± 16.67) than those with less than 2 h (72.00 ± 20.12) or 2–4 h (72.00 ± 19.52) of standing time. Participants with less attention to foot health reported more difficulty in footwear choice (55.80 ± 22.28) compared to with higher attention (63.65 ± 21.44).

General foot health showed significant differences according to daily standing time (F = 3.74, *p* = .006), foot self-care (t = 3.57, *p* < .001), and current foot discomfort (t=-6.84, *p* < .001). Post-hoc analysis revealed that general foot health was poorer in participants who stood for 6–8 h per day (62.00 ± 17.42) compared to those who stood for less than 2 h (76.00 ± 16.31). Interestingly, participants who reported without foot self-care (70.52 ± 15.30) had poorer general foot health than those who reported with foot self-care (73.00 ± 19.42). Participants experiencing current foot discomfort (48.33 ± 11.15) also demonstrated poorer general foot health compared to those without discomfort (72.09 ± 17.14) (Table 4).

**Table 4:** Differences of stress and foot health status by general characteristics of participants (*N* = 184)

Characteristics	Categories	Stress			Foot pain			Foot function			Easiness of footwear choice			General foot health		
		Mean	± SD	t or F (p) Scheffe	Mean	± SD	t or F (p) / Scheffe	Mean	± SD	t or F (p) Scheffe	Mean	± SD	t or F (p) Scheffe	Mean	± SD	t or F (p) Scheffe
Gender	Female	27.31	± 3.42	-0.02(0.988)	83.73	± 14.69	0.53(0.599)	81.57	± 18.03	-0.10(0.922)	65.02	± 21.15	-1.04(0.301)	70.67	± 17.21	0.20(0.844)
	Male	27.32	± 4.08		82.21	± 17.55		81.91	± 21.14		69.22	± 21.96		70.00	± 20.45	
Age	≤ 25^1^	27.32	± 3.87	0.62(0.606)	83.43	± 16.45	0.18(0.912)	80.98	± 19.54	0.62(0.604)	68.19	± 21.19	2.98(0.033)	71.89	± 18.38	1.56(0.200)
	26–30^2^	27.19	± 2.56		83.71	± 13.23		85.48	± 16.50		64.73	± 21.24	1 > 4	68.39	± 18.28	
	31–35^3^	28.60	± 3.41		86.00	± 11.97		78.00	± 17.19		60.00	± 16.03		73.00	± 16.36	
	≥ 36^4^	26.69	± 2.30		81.56	± 10.44		81.56	± 15.57		52.50	± 21.20		62.50	± 10.00	
Academic year	1st^1^	26.40	± 3.30	3.62(0.014)^*^	80.12	± 18.46	1.39(0.249)	83.45	± 16.77	1.65(0.180)	69.21	± 21.03	1.60(0.191)	70.71	± 19.18	1.23(0.301)
	2nd^2^	26.23	± 3.65	4 > 1	87.14	± 14.82		86.00	± 16.08		68.57	± 21.18		75.14	± 17.38	
	3rd^3^	27.98	± 3.53		83.21	± 14.41		81.42	± 18.72		66.42	± 21.80		70.19	± 17.26	
	4th^4^	28.07	± 3.41		83.89	± 13.16		77.59	± 20.83		60.74	± 20.75		67.78	± 17.34	
Daily standing time (hr)	< 2^1^	26.94	± 2.91	0.33(0.856)	91.86	± 11.25	5.70(< 0.001)^*^	92.43	± 12.80	5.04(0.001)^*^	72.00	± 20.12	3.60(0.008)^*^	76.00	± 16.31	3.74(0.006)^*^
	2–4^2^	27.10	± 2.92		84.00	± 13.31	1 > 4	82.75	± 16.37	1 < 2,3	72.00	± 19.52	1,2 > 4	74.00	± 15.82	1 > 4
	4–6^3^	27.29	± 3.66		83.08	± 13.22		79.33	± 18.81		63.85	± 21.38		70.38	± 18.47	
	6–8^4^	27.80	± 3.79		75.88	± 19.87		74.63	± 19.56		57.00	± 16.67		62.00	± 17.42	
	> 8^5^	27.53	± 5.09		83.82	± 11.39		80.29	± 22.11		65.10	± 29.68		71.76	± 18.45	
Clinical practice period (month)	No experience^1^	26.32	± 3.44	3.60(0.014)^*^	83.31	± 17.16	0.03(0.992)	84.61	± 16.40	2.20(0.089)	68.92	± 20.96	2.12(0.100)	72.73	± 18.40	1.64(0.183)
	< 6^2^	27.86	± 3.37	4 > 1	84.64	± 9.30		87.50	± 11.05		73.33	± 18.67		76.43	± 14.99	
	< 12^3^	28.00	± 3.49		83.30	± 15.29		78.86	± 20.23		62.12	± 21.89		67.50	± 16.86	
	≥ 12^4^	28.10	± 3.52		83.47	± 13.55		77.76	± 21.09		62.04	± 21.27		68.16	± 17.99	
Shoes worn during clinical practice^†^	Sneaker type	27.79	± 3.66	3.70(0.313)	84.09	± 11.42	0.66(0.576)	84.09	± 17.07	2.16(0.094)	63.03	± 19.87	1.09(0.357)	69.70	± 16.10	0.96(0.411)
	Sandal type	28.10	± 3.43		84.10	± 14.97		77.54	± 21.02		64.28	± 22.16		69.25	± 17.52	
	Others	28.43	± 3.10		75.71	± 9.76		76.43	± 18.64		59.05	± 22.91		62.86	± 21.38	
Shoe purchase method^†^	Bulk purchase	28.07	± 3.38	5.46(0.105)	83.01	± 14.31	0.24(0.790)	79.70	± 20.14	1.74(0.178)	62.25	± 21.22	2.14(0.121)	69.16	± 18.09	1.02(0.364)
	Individual purchase	27.88	± 3.78		85.42	± 11.60		78.75	± 19.01		68.06	± 21.71		68.33	± 14.35	
Preferred shoe type	Sneakers	27.09	± 3.49	0.89(0.413)	84.65	± 13.86	2.40(0.094)	82.24	± 18.09	0.56(0.570)	66.77	± 21.66	1.06(0.348)	71.34	± 17.29	0.91(0.405)
	Slippers	27.77	± 3.79		82.08	± 17.77		81.15	± 20.51		65.00	± 20.24		69.79	± 19.40	
	Others	28.11	± 2.80		73.89	± 16.73		75.56	± 15.09		56.30	± 21.63		63.33	± 15.81	
Preferred heel height (cm)	< 3	27.27	± 3.56	-0.31(0.756)	82.39	± 15.93	-1.64(0.102)	81.59	± 18.58	-0.05(0.964)	65.41	± 21.98	-0.43(0.671)	70.29	± 18.08	-0.33(0.739)
	≥ 3	27.46	± 3.51		86.63	± 12.47		81.74	± 18.80		66.96	± 19.32		71.30	± 17.08	
Preferred shoe size	Larger	27.06	± 3.24	0.39(0.679)	85.63	± 13.74	1.75(0.178)	80.38	± 19.53	0.69(0.504)	65.00	± 21.75	0.73(0.486)	71.00	± 17.33	0.89(0.413)
	Same	27.50	± 3.80		81.58	± 16.08		82.33	± 17.94		66.01	± 21.03		69.80	± 18.22	
	Smaller	28.00	± 2.65		88.33	± 20.21		91.67	± 14.43		80.00	± 20.00		83.33	± 15.28	
Foot self-care	Yes	27.62	± 4.13	2.97(0.003)^*^	82.67	± 18.40	4.33(< 0.001)^*^	80.08	± 20.55	3.48(0.001)^*^	68.78	± 22.08	1.25(0.211)	73.00	± 19.42	3.57(< 0.001)^*^
	No	27.31	± 3.21		84.18	± 13.28		83.61	± 17.20		66.74	± 19.92		70.52	± 15.30	
Attention to foot health	Low^1^	26.67	± 3.29	0.67(0.514)	82.59	± 14.37	0.23(0.794)	77.96	± 18.62	1.29(0.279)	55.80	± 22.28	3.76(0.025)	65.19	± 21.55	1.81(0.166)
	Moderate^2^	26.62	± 3.26		87.70	± 13.19		85.95	± 16.15		67.60	± 21.13	1 > 3	74.70	± 17.95	
	High^3^	28.14	± 3.70		78.39	± 15.98		76.49	± 20.03		63.65	± 21.44		65.60	± 16.38	
Experience using foot care facilities	Yes	29.05	± 3.78	2.28(0.024)	82.89	± 11.46	-0.17(0.867)	82.63	± 16.61	0.25(0.805)	64.56	± 22.89	-0.27(0.790)	66.84	± 18.87	-0.96(0.340)
	No	27.12	± 3.47		83.52	± 15.62		81.52	± 18.84		65.94	± 21.18		70.97	± 17.68	
Current foot discomfort	Yes	27.50	± 3.55	0.19(0.852)	70.00	± 15.23	-3.25(0.001)^*^	72.50	± 17.65	-1.77(0.078)	60.00	± 20.50	-0.98(0.331)	48.33	± 11.15	6.84(< 0.001)^*^
	No	27.30	± 3.55		84.39	± 14.81		82.27	± 18.53		66.20	± 21.36		72.09	± 17.14	

^†^No experience in clinical practice were excluded from this section

## Discussion

One of the most notable findings of this study was an apparently contradictory pattern in nursing students’ foot health status and associated characteristics. Nursing students who reported using methods for relieving foot pain showed more severe foot pain and poorer foot function compared to those who did not use such methods. This seemingly paradoxical relationship requires careful interpretation. Two possible explanations exist for these findings. First, nursing students might have initiated foot self-care methods in response to existing foot problems, suggesting that foot discomfort preceded foot self-care practices. This aligns with recent research demonstrating that healthcare professionals often recognize the importance of foot health only when problems arise [20]. Alternatively, Nursing students who actively engage in foot self-care might be more aware of and sensitive to their foot conditions, leading to more detailed reporting of pain and functional limitations.

This study revealed that 93.5% of nursing students reported foot discomfort, with various foot-related diseaes. conditions including toenail disorders, tinea pedis, eczema, plantar fasciitis, and plantar keratosis. Bernardes et al. (2023) recently demonstrated that prolonged standing environments significantly impact nursing students’ foot health, particularly during clinical practice periods [10]. This finding is supported by Davis and Kotowski (2015), who identified high prevalence rates of lower extremity disorders among healthcare professionals [21]. This study found that nursing students’ foot pain scores averaged 83.52 ± 15.21 points and foot function scores 81.64 ± 18.63 points, with those standing for 6–8 h per day experiencing more severe foot pain compared to those standing less than 2 h. These findings align with Reed et al. (2014) who identified prolonged standing as a significant risk factor for foot and ankle disorders among healthcare professionals [8].

The relationship between clinical practice experiences and foot health deserves particular attention. This study showed that nursing students with more clinical practice experience reported poorer foot health outcomes, consistent with previous research on occupational foot health in healthcare settings [22]. This suggests that foot health problems may accumulate with increased exposure to clinical environments, highlighting the need for early intervention strategies.

Recent comprehensive findings from Davis and Kotowski (2015) who analyzed 132 studies and found that MSD in healthcare professionals are not limited to the commonly studied low back pain, but also significantly affect lower extremities, with annual prevalence rates of 36.0% [21]. Their review particularly emphasized that as healthcare practices evolve with changes such as no-lift policies and increasing use of mechanical aids, the pattern of MSD may shift from traditional low back issues to other body regions. This suggests that preventive strategies for nursing students should consider not only back health but also comprehensive musculoskeletal health including foot care.

Based on these findings, it is crucial to develop and implement foot health care education programs for nursing students. Recent research emphasizes that early preventive interventions during nursing education can significantly impact long-term foot health outcomes [9, 23]. Such education should be implemented before clinical practice begins, covering the importance of foot health, common foot problems, preventive care methods, and appropriate footwear selection. This aligns with recommendations from recent occupational health research highlighting the importance of early preventive strategies in healthcare professions [9, 20].

The clinical implications of our findings extend beyond individual nursing student health. As Davis and Kotowski (2015) demonstrate through their comprehensive review, MSD among healthcare professionals can significantly affect both work performance and QoL [21]. This study indicates that nursing students’ preference for sneaker-type shoes and low heels (less than 3 cm) are positive factors for foot health [13]. However, 45.5% of nursing students chose their clinical practice shoes through bulk purchasing, which fails to account for individual foot health needs. Anderson et al. (2017) emphasized the importance of considering individual characteristics when selecting occupational footwear, suggesting that institutional bulk purchasing practices may need reconsideration [24].

This study adds to the growing body of evidence highlighting the importance of foot health in nursing education and practice. However, we acknowledge several contextual factors that may influence our findings. The relationship between standing time and foot health outcomes may be affected by various personal factors not captured in this study, such as body mass index, pre-existing medical conditions, and activities outside clinical practice. Additionally, the timing of data collection relates to clinical practice sessions and the specific pattern of standing time (daily versus intermittent) may influence participants’ responses to foot health assessments. Future research directions should build upon these findings by conducting longitudinal studies to track changes in foot health throughout nursing education and early career practice [10]. Additionally, intervention studies examining the effectiveness of various preventive strategies and educational programs are needed, as suggested by current systematic reviews in healthcare settings [23].

### Study limitations

This study has several limitations. First, we did not exclude participants with pre-existing musculoskeletal or rheumatological conditions, prior foot disorders, or those engaged in competitive sports activities. These factors could have influenced the assessment of foot health status. Future studies should consider controlling these potential confounding variables to better isolate the effects of clinical practice on nursing students’ foot health.

Second, the Participants were limited to nursing students from universities in B metropolitan city and K province, which may not be representative of the broader nursing student population in South Korea. This geographical limitation could affect the generalizability of our findings.

Third, the use of an online survey platform for data collection, while efficient, has inherent limitations. Participants’ self-reported responses might be subject to recall bias, and the online format may have excluded potential participants who had limited access to or were less comfortable with digital platforms.

Finally, the cross-sectional nature of this study prevents us from establishing causal relationships between clinical practice experiences and foot health outcomes. Longitudinal studies would be valuable to track changes in foot health status throughout nursing students’ clinical practice periods and to better understand the temporal relationship between practice experiences and foot health.

### Future research

Several directions for future research emerge from this study’s findings and limitations. First, longitudinal studies tracking nursing students from admission through graduation are needed to understand the progression of foot health and their relationship with clinical practice experiences, as emphasized by recent research [10]. Such studies should control pre-existing conditions and confounding variables identified in our limitations.

Second, intervention studies should be conducted to develop and evaluate the effectiveness of foot health education programs [23]. These programs should be tailored to different academic years and clinical practice periods, with particular emphasis on preventive care strategies before the onset of foot health problems.

Third, research is needed to evaluate the impact of footwear choices and institutional purchasing practices on foot health outcomes, building upon recent occupational health research [8]. This could include comparative studies of different shoe types and assessment of individual fitting versus bulk purchasing approaches.

Finally, qualitative research exploring nursing students’ experiences, perceptions, and decision-making processes regarding foot health care could provide valuable insights for developing more effective interventions and educational programs, as suggested by current healthcare workforce studies [20].

## Conclusions

This study investigated the foot health status and associated characteristics of student nursing nurses. Most student nurses reported foot discomfort, with stress and foot health disorders worsening as they progressed through their academic years and gained more clinical practice period. Additionally, longer daily standing times were associated with more severe foot pain and decreased foot function.

These findings underscore the critical importance of proactive foot health care for nursing students. Early recognition and intervention are key to preventing progressive foot-related disorders. Nursing student should be educated about the significance of foot self-care and equipped with knowledge and strategies to protect their foot health before symptoms escalate. This includes understanding the importance of appropriate footwear selection and seeking timely medical intervention when discomfort emerges.

The comprehensive approach to foot health demonstrated in this study provides foundational evidence for developing targeted educational interventions. By addressing these health concerns early, we can potentially enhance occupational health, QoL, and ultimately, the quality of nursing care. Our findings contribute to a deeper understanding of foot-health challenges faced by nursing students and emphasize the critical need for proactive health management in nursing education.

## Data Availability

The datasets used in this study are available from the corresponding author upon reasonable request.
